# Cerebrovascular Reactivity and Central Arterial Stiffness in Habitually Exercising Healthy Adults

**DOI:** 10.3389/fphys.2018.01096

**Published:** 2018-08-17

**Authors:** Kathleen B. Miller, Anna J. Howery, Ronée E. Harvey, Marlowe W. Eldridge, Jill N. Barnes

**Affiliations:** ^1^Bruno Balke Biodynamics Laboratory, Department of Kinesiology, University of Wisconsin–Madison, Madison, WI, United States; ^2^Mayo Clinic College of Medicine and Science, Mayo Clinic, Rochester, MN, United States; ^3^Division of Critical Care, Department of Pediatrics, University of Wisconsin School of Medicine and Public Health, Madison, WI, United States; ^4^John Rankin Laboratory of Pulmonary Medicine, University of Wisconsin School of Medicine and Public Health, Madison, WI, United States

**Keywords:** aging, cerebral blood flow, aerobic exercise, pulse wave velocity, blood pressure

## Abstract

Reduced cerebrovascular reactivity to a vasoactive stimulus is associated with age-related diseases such as stroke and cognitive decline. Habitual exercise is protective against cognitive decline and is associated with reduced stiffness of the large central arteries that perfuse the brain. In this context, we evaluated the age-related differences in cerebrovascular reactivity in healthy adults who habitually exercise. In addition, we sought to determine the association between central arterial stiffness and cerebrovascular reactivity. We recruited 22 young (YA: age = 27 ± 5 years, range 18–35 years) and 21 older (OA: age = 60 ± 4 years, range 56–68 years) habitual exercisers who partake in at least 150 min of structured aerobic exercise each week. Middle cerebral artery velocity (MCAv) was recorded using transcranial Doppler ultrasound. In order to assess cerebrovascular reactivity, MCAv, end-tidal carbon dioxide (ETCO_2_), and mean arterial pressure (MAP) were continuously recorded at rest and during stepwise elevations of 2, 4, and 6% inhaled CO_2_. Cerebrovascular conductance index (CVCi) was calculated as MCAv/MAP. Central arterial stiffness was assessed using carotid–femoral pulse wave velocity (PWV). Older adults had higher PWV (YA: 6.2 ± 1.2 m/s; OA: 7.5 ± 1.3 m/s; *p* < 0.05) compared with young adults. MCAv and CVCi reactivity to hypercapnia were not different between young and older adults (MCAv reactivity, YA: 2.0 ± 0.2 cm/s/mmHg; OA: 2.0 ± 0.2 cm/s/mmHg; *p* = 0.77, CVCi reactivity, YA: 0.018 ± 0.002 cm/s/mmHg^2^; OA: 0.015 ± 0.001 cm/s/mmHg^2^; *p* = 0.27); however, older adults demonstrated higher MAP reactivity to hypercapnia (YA: 0.4 ± 0.1 mmHg/mmHg; OA: 0.7 ± 0.1 mmHg/mmHg; *p* < 0.05). There were no associations between PWV and cerebrovascular reactivity (range: *r* = 0.00–0.39; *p* = 0.07–0.99). Our results demonstrate that cerebrovascular reactivity was not different between young and older adults who habitually exercise; however, MAP reactivity was augmented in older adults. This suggests an age-associated difference in the reliance on MAP to increase cerebral blood flow during hypercapnia.

## Introduction

A healthy brain is highly sensitive to changes in arterial carbon dioxide (CO_2_) such that elevations in the arterial partial pressure of carbon dioxide (P_a_CO_2_) can cause profound vasodilation of cerebral vasculature and hypercapnia is associated with augmentation of global cerebral blood flow (CBF) (Kety and Schmidt, 1948). Measuring the CBF response to hypercapnia is often used to test cerebrovascular function and is termed cerebrovascular reactivity (Iliff et al., 1974). Importantly, cerebrovascular reactivity is lower in individuals with a history of stroke, patients with dementia or Alzheimer’s disease, and may be a biomarker of risk of cognitive decline (Iadecola, 2004; Silvestrini et al., 2006; Glodzik et al., 2013).

As advancing age increases the risk for development of cerebrovascular disease and cognitive decline (Choi et al., 1998), it is important to distinguish physiological or primary aging from pathology. There are conflicting reports regarding the changes in cerebrovascular reactivity to hypercapnia related to primary aging with some studies demonstrating age-associated declines (Rogers et al., 1985; Reich and Rusinek, 1989; Tsuda and Hartmann, 1989; Matteis et al., 1998; Barnes et al., 2012), some demonstrating age-associated increases (Zhu et al., 2013) while others show no age-associated declines (Schieve and Wilson, 1953; Schwertfeger et al., 2006; Galvin et al., 2010; Oudegeest-Sander et al., 2014; Coverdale et al., 2017). These discrepancies in the literature may be due to methodological differences as well as participants’ underlying comorbidities, biological sex, medication use, or habitual exercise status. For example, the aforementioned studies reported a variety of exercise participation from sedentary to recreationally active. Previous studies have shown a positive relationship between cardiorespiratory fitness and cerebrovascular reactivity to hypercapnia (Bailey et al., 2013; Barnes et al., 2013; Murrell et al., 2013); thus, it is important to consider activity status of participants as the reported effects of aging may have been amplified by sedentary behavior.

Early studies investigating age-associated differences in cerebrovascular reactivity to hypercapnia did not take into account the blood pressure response during CO_2_ inhalation (Schieve and Wilson, 1953; Rogers et al., 1985; Reich and Rusinek, 1989; Tsuda and Hartmann, 1989; Matteis et al., 1998; Schwertfeger et al., 2006; Galvin et al., 2010). Because changes in perfusion pressure can influence CBF during hypercapnia (Aaslid et al., 1989; Perry et al., 2014), higher mean arterial pressure (MAP) could augment CBF in the absence of cerebral microvessel vasodilation (Claassen et al., 2007). Thus, when MAP is not accounted for, it is difficult to determine the magnitude of cerebral vasodilatory responses, which is important for adequately matching cerebral perfusion with neuronal metabolic demand.

Additionally, cerebral vasodilatory responses could be affected by age-related changes in central arterial stiffness. The structure and function of the central elastic arteries are associated with microvascular health and tissue function (Mitchell et al., 2011). Central elastic arteries supply the brain with blood flow and have an important role in dampening the high-pressure fluctuations emitted during myocardial contraction. Arterial stiffness in the central arteries may allow high pressure fluctuations to be translated into the cerebral microvessels and impair their function (O’Rourke and Safar, 2005; Mitchell, 2008; London and Pannier, 2010; Tuttolomondo et al., 2010). Central arterial stiffness increases with advancing age but is lower in adults who engage in habitual exercise compared with age-matched sedentary adults (Vaitkevicius et al., 1993; Tanaka et al., 1998, 2000).

Despite evidence that elevated central arterial stiffness can impact cerebrovascular function, information regarding the relationship between central arterial stiffness and cerebrovascular reactivity in healthy adults is limited. In this context, the purpose of our cross-sectional study was twofold. First, we sought to evaluate cerebrovascular reactivity to hypercapnia in young and older habitually exercising active adults while taking into account changes in perfusion pressure. We hypothesized that there would be no age-associated reduction in cerebrovascular reactivity in healthy adults who habitually exercise. Second, we sought to evaluate the association between central arterial stiffness and cerebrovascular reactivity. We hypothesized that cerebrovascular reactivity would be inversely related to central arterial stiffness in both young and older adults.

## Materials and Methods

### Participants

Habitually exercising adults were recruited via recruitment flyers and word of mouth. Forty-three adults including 21 older adults and 22 young adults participated in the study. All participants performed at least 150 min of structured moderate intensity aerobic exercise per week in their free time. Individuals who did not meet these criteria were excluded from the study. Participants were also non-smoking, had a body mass index (BMI) <34 kg/m^2^, did not have a clinical diagnosis of hypertension, were not taking antihypertensive medication, had seated brachial blood pressure measurement <140/90 during screening, and were excluded if they presented with: history or evidence of hepatic, renal, or hematological disease, peripheral vascular disease, stroke or neurovascular disease, cardiovascular disease, diabetes, or other chronic pathologies (as determined by a health questionnaire). Participants were not taking antihypertensive medication or any other vasoactive medications other than statins (older adults *n* = 2) or thyroid medication (older adults *n* = 4). Young women were studied during days 2–6 of their menstrual cycle or during the non-active pill phase of oral contraceptives (*n* = 7). Young women were not pregnant which was confirmed by a urine pregnancy test. Older women must have been postmenopausal for at least 1 year and not taking menopausal hormone therapy. All study procedures were approved by the Institutional Review Board of the Mayo Clinic and the University of Wisconsin–Madison and were performed according to the Declaration of Helsinki, including obtaining written informed consent from each participant.

### Experimental Procedures

Experimental study days were completed at Mayo Clinic (*n* = 13) and the Bruno Balke Biodynamics Laboratory at the University of Wisconsin–Madison (*n* = 30). A mix of young and older participants were studied at each site. Study protocols were identical at the two sites. The tests were conducted in controlled ambient temperature between 22 and 24°C. Central arterial stiffness measurements and stepped hypercapnia trials were performed on a separate study visit from the 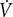
O_2max_ test. Prior to the study visits, participants were asked to fast overnight, to abstain from non-steroidal anti-inflammatory drugs (NSAIDs) for 5 days, and to abstain from caffeine, exercise, and alcohol for 24 h. Additionally, participants did not take any over the counter medications, vitamins or supplements on the day of the study visit. Upon arrival, height and weight were measured using a standard scale. BMI was calculated as kg/m^2^.

### Habitual Aerobic Exercise Participation and Maximal Aerobic Capacity

Habitual aerobic exercise participation was assessed with a 7 day exercise log and the GODIN questionnaire. The GODIN questionnaire is a 4-item questionnaire that describes the number of times during a typical week one engages in mild, moderate, and strenuous leisure-time physical activity, as detailed in Godin and Shephard (1985). Maximal aerobic capacity (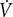
O_2max_) was performed on an incremental cycle ergometer test to exhaustion (Lode B.V., Groningen, Netherlands). During the exercise test, ventilation and gas exchange variables were measured utilizing a metabolic cart (Parvo Medics, Sandy, UT, United States). Participants maintained a self-selected pace. After a 1 min warm up, the workload increased by 20–40 W (depending on the individual’s age and sex) each minute until acceptable test criteria was met. The criteria for an acceptable test was failure to increase VO_2_ with an increasing workload, a respiratory exchange ratio (RER) of over 1.10, and a maximum heart rate (HR) value within 10 bpm of the age predicted maximum (220 – age). All participants met the criteria for an acceptable 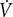
O_2max_ test.

### Instrumentation

After 10 min of supine rest, baseline MAP was taken in triplicate with a non-invasive brachial blood pressure cuff in the supine position (Datex Ohmeda, GE Healthcare, Fairfield, CT, United States). The average of the three measurements is reported. Participants were instrumented with a three-lead electrocardiogram to continually monitor HR and a pulse oximeter to monitor oxygen saturation (SpO_2_) (Datex Ohmeda, GE Healthcare, Fairfield, CT, United States). Breath-by-breath end-tidal carbon dioxide (ETCO_2_) was continually measured and recorded (Datex Ohmeda, GE Healthcare, Fairfield, CT, United States). A non-invasive finger blood pressure cuff placed around the middle finger continually measured and recorded beat-by-beat MAP (Finapres Medical Systems, Amsterdam, Netherlands; Nexfin, Edwards Lifesciences, Irvine, CA, United States). A height-correcting unit was used in order to account for any differences between the height of the finger and the height of the heart.

### Central Arterial Stiffness

Carotid–femoral pulse wave velocity (PWV) measurements were completed utilizing arterial tonometry as previously reported (Harvey et al., 2016). Briefly, after 15 min of supine rest, high-fidelity pressure waveforms were recorded for at least 10 heartbeats non-invasively using a pencil-type Millar Micro-tip pressure transducer from the carotid and femoral arteries and PWV was calculated using the intersection tangent foot-to-foot algorithm (i.e., the delay in arrival of the arterial pressure waveform between the carotid and femoral sites, relative to the QRS complex) as described previously (O’Rourke and Gallagher, 1996; Wilkinson et al., 1998) (Sphygmocor, AtcorMedical, Sydney, Australia). The average of three to five trials obtained in succession is reported. Distance was defined as the distance between the common carotid and femoral recording sites minus the distance of the common carotid recording site to the suprasternal notch. Tonometry transit distance from the carotid pulse site, the suprasternal notch, and the femoral pulse site was measured manually with a tape measure.

### Middle Cerebral Artery Velocity

Participants were imaged using a 2 MHz Transcranial Doppler (TCD) probe (Neurovision System, Multigon, Yonkers, NY, United States and Spencer Technologies, Redmond, WA, United States) to estimate right middle cerebral artery velocity (MCAv) (Bishop et al., 1986; Poulin and Robbins, 1996). The 2 MHz probe was placed over the temporal bone of the skull just above the zygomatic arch between the frontal process and front of the ear. The probe was secured with a headband in order to maintain optimal insonation position and angle throughout the study protocol.

### Stepped Hypercapnic Trials

Stepped hypercapnia trials were performed as previously described using a steady-state, open-circuit technique (Berkenbosch et al., 1989; Xie et al., 2006; Barnes et al., 2012, 2013). Briefly, participants were in the supine position and fitted with a mask covering their nose and mouth containing a one-way valve to prevent re-breathing (Hans Rudolph Inc., Shawnee, KS, United States). After breathing room air, stepwise elevations of 2, 4, and 6% inspired CO_2_, 21% oxygen, and balanced nitrogen were administered. ETCO_2_ was elevated and maintained constant for 3 min at each level of inspired CO_2_.

### Data Analysis and Statistics

Data were collected at 250 Hz and analyzed off-line using signal processing software (WinDaq, DATAQ Instruments, Akron, OH, United States). Participant demographics and baseline characteristics were compared using a one-way ANOVA. Beat-by-beat hemodynamic measurements were averaged over the final minute of room air breathing and at each level of hypercapnia. Cerebrovascular reactivity was quantified as the linear relationship between MCAv and ETCO_2_ during stepped hypercapnia. In order to account for changes in perfusion pressure that may affect flow, cerebrovascular conductance index (CVCi) was calculated as MCAv/MAP and also related to changes in ETCO_2_ during hypercapnia. MAP reactivity was calculated as the linear relationship between MAP and ETCO_2_. Cerebrovascular reactivity and MAP reactivity slopes were compared between young and older adults using a one-way ANOVA. A two-way repeated measures ANOVA compared the cerebral hemodynamic and gas exchange variables during stepped hypercapnia followed by the Holm-Sidak method to test pairwise comparisons. The associations between central arterial stiffness and cerebrovascular reactivity were evaluated utilizing a Spearman rank order correlation in separate groups (young and older participants). In a secondary analysis, we employed multivariable linear regression with MCAv as the dependent variable and MAP and ETCO_2_ as the independent variables in separate groups (young and older adults) in order to determine the contribution of the linear combination of MAP and ETCO_2_ to MCAv. Statistical significance was set a priori at *p* < 0.05. In our previous study, it was found that CVCi reactivity was statistically different between young (*n* = 12) and older (*n* = 10) adults (1.0 ± 0.2 cm/s/mmHg^2^ vs. 0.6 ± 0.1 cm/s/mmHg^2^, respectively, *p* < 0.05) (Barnes et al., 2012). Based on this data, we determined that if a significant difference in CVCi reactivity would be present, a sample-size of *n* = 16 subjects (8 per group) would provide a statistical power of 80% to detect a difference at α = 0.05. However, because we included the potential association between central arterial stiffness and cerebrovascular reactivity, we recruited at least 20 subjects per group.

## Results

Participant characteristics are displayed in **Table 1**. Young and older adults had a similar BMI and supine systolic blood pressures at baseline, yet older adults had higher supine diastolic blood pressures and MAP when compared with young adults. Additionally, older adults demonstrated higher carotid–femoral PWV indicating higher central arterial stiffness. The coefficient of variation for PWV trials was 9%. Young and older adults had similar aerobic exercise participation based on the GODIN questionnaire and MET minutes per week calculated from the 7-day recall of exercise (**Table 1**).

**Table 1:** Characteristics of participants.

Variable	Young adults (*N* = 22)	Older adults (*N* = 21)	*p*-Value
Male/female (*n*)	10/12	10/11	
Age (years)	27 ± 5	60 ± 4	**<0.001**
Height (cm)	172 ± 8	162 ± 8	0.35
Weight (kg)	68 ± 10	65 ± 15	0.95
Body mass index (kg/m^2^)	23 ± 2	22 ± 4	0.46
Heart rate at rest (bpm)	51 ± 6	51 ± 6	0.60
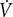 O_2max_ (ml/kg/min)	47 ± 6	35 ± 6	**<0.001**
GODIN questionnaire	67 ± 20	66 ± 28	0.84
MET minutes per week	4088 ± 1580	4688 ± 3414	0.31
Systolic blood pressure (mmHg)	117 ± 10	116 ± 13	0.15
Diastolic blood pressure (mmHg)	68 ± 7	72 ± 7	**0.004**
Mean arterial pressure (mmHg)	84 ± 7	87 ± 9	**0.01**
Carotid–femoral pulse wave velocity (m/s)	6.2 ± 1.2	7.5 ± 1.3	**<0.001**
MCAv at rest (cm/s)	63 ± 17	55 ± 15	0.15

*Demographic data are mean ± standard deviation. MET, metabolic equivalent; MCAv, middle cerebral artery velocity. Bolded *p*-values are *p* < 0.05 compared to young adults (one-way ANOVA)*.

**FIGURE 1:**
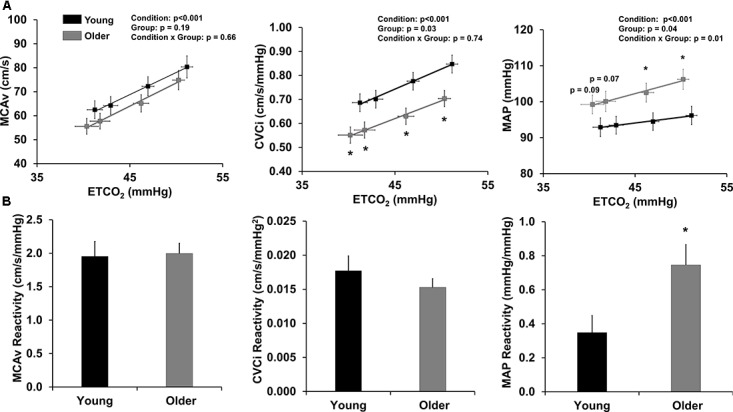
Cerebrovascular reactivity to hypercapnia. **(A)** Relationship between end-tidal CO_2_ (ETCO_2_) and variables of interest: MCAv (left), CVCi (middle), and MAP (right) during hypercapnia. Data are mean ± standard error of the mean. CVCi = MCAv/MAP. ^∗^*p* < 0.05 compared to young adults (two-way repeated measures ANOVA). **(B)** Reactivity slopes for MCAv (left), CVCi (middle), and MAP (right) during hypercapnia. Young adults are shown in black and older adults are shown in gray. Data are mean ± standard error of the mean. ^∗^*p* < 0.05 compared to young adults (one-way ANOVA).

Cerebral hemodynamic and gas exchange variables during the stepped hypercapnia protocol are displayed in **Figure 1A**. SpO_2_ was 99% during room air inhalation and did not change during the protocol or differ between groups (data not shown). MCAv was not different between groups at any level; however, older adults demonstrated lower CVCi at room air and during each level of stepped hypercapnia (**Figure 1A**). Older adults also demonstrated higher MAP than young adults at 4 and 6% CO_2_ (**Figure 1A**).

Cerebrovascular reactivity to stepped hypercapnia is displayed in **Figure 1B**. MCAv and CVCi reactivity to stepped hypercapnia were not different between young and older adults; however, older adults demonstrated a significantly higher MAP reactivity compared to young adults (**Figure 1B**). Cerebrovascular reactivity did not differ by study site (data not shown).

When examining the association between central arterial stiffness and cerebrovascular reactivity, there were no correlations in young or older adults (**Table 2**). There was a trend for a positive correlation between carotid–femoral PWV and MAP reactivity in young adults such that young adults with higher central arterial stiffness demonstrated a greater MAP response to hypercapnia (*p* = 0.07). No such association was present in older adults (**Table 2**).

**Table 2:** Associations between cerebrovascular reactivity and central arterial stiffness.

Variable	Young adults (*N* = 22)		Older adults (*N* = 21)	
Carotid–femoral pulse wave velocity and MCAv reactivity slope	*r* = 0.09	*p* = 0.68	*r* = -0.14	*p* = 0.55
Carotid–femoral pulse wave velocity and CVCi reactivity slope	*r* = -0.02	*p* = 0.92	*r* = -0.20	*p* = 0.38
Carotid–femoral pulse wave velocity and MAP reactivity slope	*r* = 0.39	*p* = 0.07	*r* = 0.00	*p* = 0.99

*Data are Spearman rank order correlations between carotid–femoral pulse wave velocity and MCAv, CVCi, and MAP reactivity slopes*.

In our multivariable linear regression analysis, only ETCO_2_ was a significant predictor of MCAv in young adults (*p* < 0.001). In older adults, both MAP and ETCO_2_ were significant predictors of MCAv (*p* < 0.001).

## Discussion

The most salient finding of the present study is that there were no age-associated differences in MCAv or CVCi reactivity to hypercapnia in healthy adults who habitually exercise. Importantly, despite no differences in MCAv responses to hypercapnia, MAP reactivity was augmented with aging. This suggests that healthy older adults may rely on a different mechanism (i.e., increase in perfusion pressure) to sufficiently increase CBF during a vasodilatory stimulus such as hypercapnia. Additionally, we report that central arterial stiffness was elevated in healthy older adults who habitually exercise compared to young adults. Contrary to our hypothesis, central arterial stiffness was not associated with measures of cerebrovascular reactivity or MAP reactivity. This finding suggests that central arterial stiffness is not associated with cerebral vasodilatory responses in healthy habitual exercisers.

This study used a cross-sectional approach to determine the effects of primary aging on cerebrovascular reactivity and its association with central arterial stiffness. It addressed many limitations of previous studies by: (1) including participants with low cardiovascular risk and excluding individuals taking vasoactive medications, (2) recruiting young and older adults with similar BMI, systolic blood pressure, and habitual exercise habits, and (3) including approximately equal distribution of men and women. In addition, this study used standard techniques to non-invasively quantify central arterial stiffness at rest and characterize cerebrovascular reactivity and beat-by-beat MAP responses during CO_2_ inhalation.

The present finding that cerebrovascular reactivity is preserved with age conflicts our previous work in healthy sedentary and recreationally active adults (Barnes et al., 2012). It is possible that the age-related differences in cerebrovascular reactivity reported previously were augmented due to the inclusion of adults who were sedentary. Although the present study did not directly compare sedentary and habitually exercising adults, the idea that habitual exercise (not necessarily intense life-long training; Zhu et al., 2013) improves or maintains cerebrovascular function has been supported by studies that have reported the association between exercise and cerebrovascular function throughout the lifespan (Ainslie et al., 2008; Bailey et al., 2013), the positive relationship between cerebrovascular reactivity and cardiorespiratory fitness (Barnes et al., 2013), and the improvement of cerebrovascular reactivity after an exercise intervention (Murrell et al., 2013). For example, Ainslie et al. (2008) found an approximately 17% lifelong increase in MCAv at rest in endurance-trained men compared to sedentary men. Another possibility for the lack of effect of age is that the older participants in our study were upper middle-aged with an average age of 60; thus, they may not have been old enough to demonstrate true “aging” effects. It has become increasingly apparent that vascular health in the middle-aged years may predict brain health (Maillard et al., 2017); therefore, we sought to study adults during midlife where interventions could be most effective.

Despite no age-related differences in MCAv or CVCi reactivity, older adults demonstrated an augmented MAP response to hypercapnia compared to young adults. Additionally, older adults demonstrated 18–20% lower CVCi during both the normocapnia and hypercapnia conditions. We also found that MAP was a predictor of MCAv in our multivariable linear regression model, only in older adults. Taken together, these findings suggest a reduced vasoreactivity of the cerebral vasculature and an augmentation of MAP during hypercapnia to elevate CBF to a level similar to young adults. Our findings are in accordance with recent studies reporting an elevated MAP response to hypercapnia in older adults compared to young adults (Claassen et al., 2007; Coverdale et al., 2017) and include one potential mechanism for this increase (i.e., central arterial stiffness). We speculate that older adults may rely on augmented perfusion pressure in response to a vasodilatory stimulus in order to compensate for some loss of cerebral microvessel function (Barnes et al., 2012) and subsequently increased MCAv to a similar magnitude as young adults. In addition, some studies have suggested that older adults demonstrate an enhanced chemoreflex in response to hypoxia (Lhuissier et al., 2012; Paleczny et al., 2014) and a decrease in CO_2_ threshold (García-Río et al., 2007) compared with young adults. It is possible that older adults had a more pronounced sympathoexcitation contributing to the higher blood pressure response. Future studies should evaluate the role of habitual exercise and aging on the chemoreflex and sympathetic nervous system response during hypercapnia.

In the present study, we report higher central arterial stiffness in older adults, as expected (Kelly et al., 1989); however, PWV values in our sample of older adults were relatively low (<7.6 m/s) (Niiranen et al., 2017). We did not find an association between cerebrovascular reactivity to hypercapnia and carotid–femoral PWV in either young or older adults with the exception of a trend for a positive association between carotid–femoral PWV and MAP reactivity in young adults. Although elevated central arterial stiffness is consistently associated with increased risk for cerebrovascular disease and cognitive decline (O’Rourke and Safar, 2005; Mitchell, 2008; Zhong et al., 2014), habitual exercise is associated with lower central arterial stiffness (Vaitkevicius et al., 1993; Tanaka et al., 2000) and is considered protective against cognitive decline (Geda et al., 2010). Because we do not see any evidence of stiffness influencing cerebrovascular function in our study, it is possible that the habitually exercising participants demonstrated an enhanced buffering capacity of the central arteries such that they are able to reduce the high-pressure fluctuations of myocardial contraction translating into the microcirculation of the brain. This idea is supported by a recent study by Tomoto et al. (2018) who reported that endurance-trained men with lower central arterial stiffness were able to more effectively buffer changes in MCA pulsatility index when stroke volume suddenly increased (during release of lower body negative pressure), compared with sedentary men. In contrast to our findings, Jaruchart et al. demonstrated that brachial-ankle PWV was negatively associated with MCA reactivity to hypercapnia in young and older healthy adults; however, brachial-ankle PWV reflects both central and peripheral hemodynamics with the inclusion of the more muscular peripheral vasculature which could augment arterial stiffness levels. In addition, this study did not report habitual exercise participation (Jaruchart et al., 2016).

The results of the present study in healthy adults could provide context for the etiology of cognitive decline in individuals with hypertension. Individuals with hypertension are at an increased risk for cerebrovascular disease and cognitive decline (Iadecola et al., 2016) and a proposed theory of the development of hypertension is that higher blood pressure is necessary to compensate and maintain adequate blood flow to the brain. For example, CBF was similar between untreated hypertensive patients and age-matched controls, whereas individuals on antihypertensive medication demonstrated reduced cerebral perfusion (Warnert et al., 2016). This suggests that elevations in MAP may be necessary for maintaining CBF. The present study found that older adults relied on increases in MAP in order to augment MCAv to the same degree as young adults when given a vasodilatory stimulus. Although the participants in the present study were not hypertensive, it is possible that even a slight increase in baseline MAP may be acting to maintain adequate blood flow to the brain and with some cases of hypertension, this is a mechanism that goes awry.

In addition, the findings of the present study are also noteworthy in understanding pathophysiology of cognitive decline. Individuals with a current diagnosis of Alzheimer’s disease demonstrate a lower cerebrovascular reactivity to hypercapnia compared with age-matched cognitively normal controls (Vicenzini et al., 2007) and Alzheimer’s patients with reduced cerebrovascular reactivity demonstrate a faster rate of cognitive decline over 1 year (Silvestrini et al., 2006). Whether or not reductions in cerebrovascular reactivity precede Alzheimer’s pathology has not been directly tested; however, it has been hypothesized that reductions in cerebrovascular function precede neuropathology (Iadecola, 2004). Reductions in cerebrovascular function not only impact neurovascular coupling but can also hinder amyloid-β washout, further expediting the disease processes (Iadecola, 2004; Kisler et al., 2017). Along these lines, the association between vascular dysfunction and other brain pathologies have been reported in middle-aged adults with varying vascular risk (Tsao et al., 2013, 2016). We have shown that elevated aortic hemodynamics were positively associated with white matter hyperintensity volume in normotensive postmenopausal women, suggesting an association between central hemodynamics and cerebrovascular damage (Barnes et al., 2017). In our population of adults who habitually exercised, we did not find a relationship between central arterial stiffness and cerebrovascular reactivity, nor did we report an age-related difference in cerebrovascular reactivity, providing one potential explanation as to why habitual exercise is protective against certain cerebral pathologies and cognitive decline. Ultimately, our study of healthy adults provides insight into primary aging and future studies can evaluate cerebrovascular responses in the context of disease.

There are several limitations to our approach. First, we are limited to a cross-sectional analysis to determine the effect of age on cerebrovascular reactivity to CO_2_, central arterial stiffness, and the association between the two. We cannot conclude that one variable causes changes in the other. Importantly, this is the first step to evaluate the potential associations before a longitudinal study evaluating aging, cerebrovascular reactivity to CO_2_, and central arterial stiffness is initiated. Second, TCD is used frequently to measure cerebral blood velocity through the MCA, an indicator of global CBF (Bishop et al., 1986; Poulin and Robbins, 1996). Using TCD to quantify CBF relies on the assumption that length of the vessel, vessel radius and blood viscosity remain constant (Serrador et al., 2000). In recent studies, the assumption that the MCA diameter does not change during a gas stimulus such as hypercapnia has been challenged (Coverdale et al., 2017); however, diameter changes were less consistent in older adult subjects compared to young adults. Third, by utilizing TCD we are limiting our CBF measurement to one vessel (MCA). Future studies should evaluate cerebrovascular reactivity in the entire cerebral circulation in order to investigate regional differences. Fourth, our population of older adults had an average age of 60 years, which is considered middle age. It is possible that because these individuals were healthy and habitually exercising, they represented a model of enhanced vascular aging that may not be representative of an average person. Finally, we attempted to recruit participants who completed at least 150 min of aerobic exercise per week; however, this data was not measured by an accelerometer. We did careful evaluation of exercise participation utilizing a questionnaire, a weekly activity log, and by evaluating cardiorespiratory fitness objectively with a 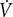
O_2max_ test. We did not, however, include young and older sedentary subjects. Future studies should directly compare the effect of aging in both sedentary and habitually active adults.

## Conclusion

In conclusion, we report no age-related differences in cerebrovascular reactivity to hypercapnia in healthy adults who habitually exercise. Importantly, older adults demonstrated an elevated MAP response to hypercapnia. In addition, we found no associations between central arterial stiffness and cerebrovascular reactivity except for a trending association between PWV and MAP reactivity in young adults. These results suggest that: (1) healthy older adults may rely on a different mechanism (i.e., increase in perfusion pressure) to sufficiently increase CBF during a vasodilatory stimulus such as hypercapnia; and (2) in healthy habitual exercisers, central arterial stiffness is not associated with cerebral vasodilatory responses. Our findings provide one potential explanation as to why habitual exercise is protective against certain cerebral pathologies and cognitive decline. Future studies should evaluate the mechanisms responsible for age-related differences in cerebral vasodilatory function, the effect of habitual aerobic exercise, and cerebrovascular reactivity in the context of disease etiology.

## Author Contributions

KM and JB designed the experiments, analyzed and interpreted the data. KM, AH, RH, ME, and JB performed the data collection. KM wrote the initial draft of the manuscript. All authors edited and approved the final draft of the manuscript.

## Conflict of Interest Statement

The authors declare that the research was conducted in the absence of any commercial or financial relationships that could be construed as a potential conflict of interest.
